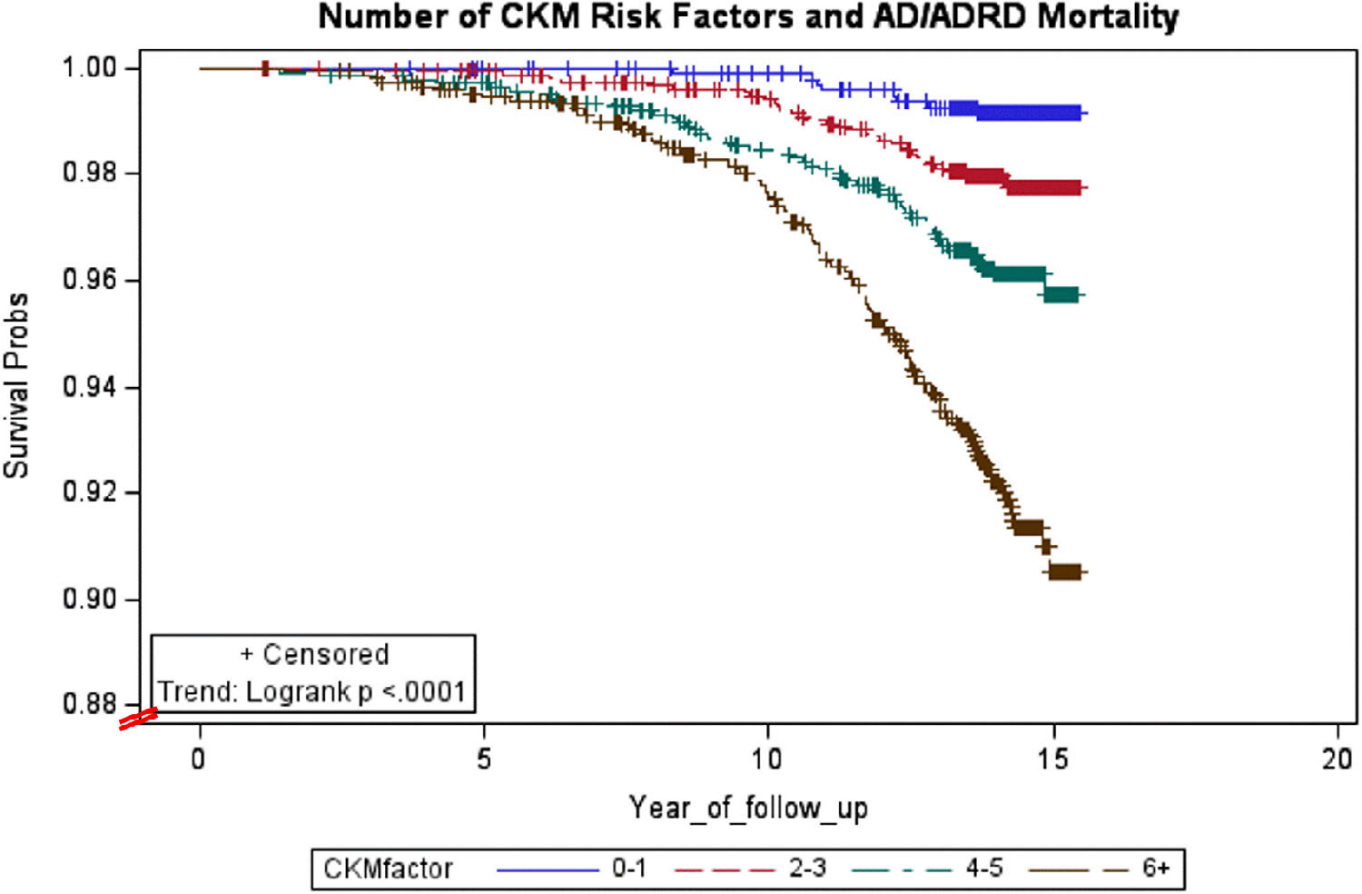# Associations between Cardiovascular‐Kidney‐Metabolic Health and the Risk of Cognitive Decline and Alzheimer’s Disease Mortality in a Multiethnic Cohort Study

**DOI:** 10.1002/alz.088022

**Published:** 2025-01-09

**Authors:** Longjian Liu, Gediminas Peter Gliebus, Xiaopeng Zhao, for the CKM‐AD/ADRD Research Group

**Affiliations:** ^1^ Drexel University Dornsife School of Public Health, Philadelphia, PA USA; ^2^ Marcus Neurosciences Institute, Baptist Health South Florida, Boca Raton, FL USA; ^3^ University of Tennessee, Knoxville, TN USA

## Abstract

**Background:**

Cardiovascular‐kidney‐metabolic (CKM) health, a term recently defined by the American Heart Association, encompasses the interplay among metabolic, chronic kidney, and cardiovascular risk factors. We aimed to investigate the predictive significance of CKM disorders with the risk of cognitive decline and Alzheimer’s disease (AD) and AD‐related dementia (ADRD) mortality in a multiethnic population.

**Method:**

We analyzed a cohort of 6,440 adults aged 45‐84 who participated in the Multiethnic Study of Atherosclerosis, with a baseline survey conducted in 2000‐2002, and were followed through to December 2015. Fourteen baseline measurements of established and emerging CKM factors were examined, including waist circumference, blood pressure (BP), carotid intimal‐medial thickness, serum glucose, triglyceride, high‐density lipoprotein cholesterol, C‐reactive protein (CRP), fibrinogen, interleukin‐6 (IL‐6), factor VIII, D‐Dimer, homocysteine, cystatin C concentrations, and urine albumin to creatinine ratio (ACR). Cognitive function was assessed using the Cognitive Abilities Screening Instrument (CASI) in 2010‐2012. AD/ADRD mortality was classified by clinical diagnoses. Logistic regression models estimated adjusted odds ratios (AOR) for cognitive decline (CASI score<Q1), and Cox regression models estimated adjusted hazard ratios (AHR) for AD/ADRD mortality. A sum CKM index reflecting the presence of multiple CKM risk factors was calculated to estimate the combined predictive effect of CKM disorders on AD/ADRD mortality risk.

**Result:**

Over a mean 13‐year follow‐up, AD/ARAD mortality rates (95%CI) were 3.6 (3.1‐ 4.3) in men (126 deaths) and 2.0 (1.6‐2.5) in women (84 deaths) per 1000‐person years. Cognitive decline was significantly associated with elevated homocysteine concentrations (AOR=1.2, 95%CI: 1.1‐1.4), and ACR (1.2, 1.03‐1.5). AD/ADRD mortality was significantly associated with high BP (AHR=1.7, 95%CI: 1.2‐2.4), and elevated glucose (1.4, 1.1‐1.9), CRP (1.4, 1.1‐1.9), IL‐6 (1.7, 1.3‐2.2), D‐Dimer (1.5, 1.2‐2.1), homocysteine (1.9, 1.4‐2.6), cystatin C (1.6, 1.2‐2.2), and ACR (2.1, 1.6‐2.8). An increased sum CKM index significantly predicted a higher risk of AD/ADRD mortality.

**Conclusion:**

This study underscores the significant role of CKM disorders in contributing to cognitive decline, with a notable predictive effect on the risk of AD/ADRD mortality. This research contributes to a growing body of evidence emphasizing the interconnectedness of metabolic, renal, and cardiovascular health with cognitive outcomes, paving the way for integrative approaches in managing these complex interrelations.